# A Quantitative Analysis of the Taxonomy of Artistic Styles

**DOI:** 10.16910/jemr.13.2.5

**Published:** 2020-06-09

**Authors:** Viviane Clay, Johannes Schrumpf, Yannick Tessenow, Helmut Leder, Ulrich Ansorge, Peter König

**Affiliations:** University of Osnabrück, Germany; University of Vienna, Austria

**Keywords:** Eye movements, eye tracking, GANs, Neural Networks, art styles

## Abstract

Classifying artists and their work as distinct art styles has been an important task of scholars in the field of art history. Due to its subjectivity, scholars often contradict one another. Our project investigated differences in aesthetic qualities of seven art styles through quantitative means. This was achieved with state-of-the-art deep-learning paradigms to generate new images resembling the style of an artist or entire era. We conducted psychological experiments to measure the behavior of subjects when viewing these new art images. Two different experiments were used: In an eye-tracking study, subjects viewed art-stylespecific generated images. Eye movements were recorded and then compared between art styles. In a visual singleton search study, subjects had to locate a style-outlier image among three images of an alternative style. Reaction time and accuracy were measured and analyzed. These experiments show that there are measurable differences in behavior when viewing images of varying art styles. From these differences, we constructed hierarchical clusterings relating art styles based on the different behaviors of subjects viewing the samples. Our study reveals a novel perspective on the classification of artworks into stylistic eras and motivates future research in the domain of empirical aesthetics through quantitative means.

## Introduction

The philosophy of aesthetics is concerned with the creation, perception, and nature of beauty. Intentionally crafted objects with an aesthetic value are commonly referred to as artworks, a subset of which are paintings. Interpreting the work of painters and their significance is a task traditionally conducted by scholars in the field of art history. These interpretations contain and are often based on, among other factors, a subjective, qualitative assessment of an artist’s work. Even though art historians can make qualitative statements about the aesthetic features that position the work of one artist in a specific era or art domain (e.g., Impressionism), these features are again the result of a subjective experience of a piece of art. This approach leaves open if there are metric properties in visual feature space that discriminate between different styles of paintings.

However, the field of empirical aesthetics aims at testing exactly this influence of the more formal, possibly quantitative or metrical characteristics. It does so by investigating formally described differences in works of art with psychological methodology (e.g., psychophysical methods). The focus of the present research is in line with this goal. We investigated differences and similarities between seven different art styles by blending quantitative manipulations and psychophysical methods.

To probe if paintings assumed to share a particular style also share metric properties that allow humans to behaviorally discriminate visual images according to these styles, we chose to apply an approach that does not require particular a priori assumptions about a specific metric property. Instead, we used deep neural networks to extract several features of art styles from a variety of different paintings classified to belong to the same style and subsequently reapplied the same extracted features to other natural images. This way, we generated novel images in the style of a painter or even an art epoch. These images were then used in two studies with human subjects. The question was if subjects seeing these images do behaviorally discriminate between the images of different styles. Humans should show discriminative behavior depending on the style of the generated images only to the extent that the deep neural networks extracted some discriminative and style-specific metric properties, as these networks could only use such metric properties to create differences between generated images.

Our first study was a visual singleton search experiment for style deviant images. Here, participants were asked to look at a screen presenting four generated images, three of which were presented in one style and one presented in another. Subjects were asked to locate the image that is different in style compared to all other images shown as accurately and quickly as possible. This is an explicit task and, thus, it might depend on some degree of the participants’ explicit knowledge of art styles. However, participants can accomplish this task also without prior knowledge to the degree that the target image of one style stands out among the distractor images of an alternative style by some underlying visual metric properties of the different styles. Related to this point, for the participants search is easier, the more the target “pops out” among the distractors by metric visual differences between target and distractor styles cf. (1) such as color or luminance. Accordingly, reaction times and accuracy of the searches were recorded. Together they provided a quantitative measure of the degree of existing visual metric property differences between art styles.

Our second study was an eye-tracking experiment where subjects viewed our generated images one by one without any explicit task. We recorded eye movements and correlated them relative to the different styles. Such a behavioral investigation provides additional style dependent measures besides the speed and accuracy of discrimination from the first task. For example, the time to dwell on locations of objects in images could be a function of their violation of expectancies (i.e., how similar an object in a generated image is to an object outside this laboratory task), such that styles that differ in their degree of realism (i.e., image-world correspondence) could differ in the overall number of locations looked at. As a singleton search task, this task does not require that our participants have explicit knowledge about the different styles. Again, to the degree that metric properties between styles varied, they could show up in behavioral differences and to the degree that different styles were more similar to one another in such properties, the behavioral differences should be smaller.

To sum up, we aimed to discover relationships in subject behavior during participants’ viewing of the seven different art styles. We constructed relationships between art styles from this quantitative data and assessed our results in the light of a similarity schema derived from art historical literature.

### Art historical literature review/background

To generate our stimuli, we selected seven art styles as training data for our deep neural networks. Four of the styles were chosen to comprise both the early and late works of the artistic movements of Impressionism and Expressionism, respectively, whereas the other three styles represent the individual styles of the artists Paul Cézanne, Paul Gauguin, and Vincent van Gogh.

Choosing Impressionism and Expressionism as the main eras for the present study proved to be of advantage, as both eras are regarded as popular due to their anecdotal striking individual stylistics, making both recognizable on their own. At the same time, the historical succession of Expressionism following Impressionism guaranteed enough commonalities between both eras to generate an area of ambiguity in which to locate the three individual artists.

Impressionism as an art era was defined by the way in which reality was represented as impressionists did not focus on representing nature in the most accurate way, but in the way they perceived it in a particular moment (2, 3). The reality depicted in paintings of impressionists can, therefore, be said to be a subjective representation of the actual reality. Tied to the structure of reality, impressionists tried to apply the broad scope of color, light, and details that constituted reality in their paintings. To achieve that, the paintings were created out of many short and ﬁne brushstrokes, like different colored pixels creating a picture on a screen, making the ﬁnal painting look more vivid and perfused by light, giving it many different shades of a sometimes-single tone.

Expressionists, on the other hand, thought that reality could only be created by accumulation of subjective ideas, thoughts, and emotions being expressed on canvas. Therefore, they tended to a rougher and broader use of color, creating shapes and forms of single tones of colors. Thereby, the realistic approach vanished gradually giving way to more unrealistic and far more abstracted structures, which represent more of the painter’s inner reality than the external reality. This inner reality of the painter became the guiding principle for expressionistic structure, as well as expressionistic coloring: Like the abstract shapes and structures given to real motifs, expressionists mainly used new and contrasting colors which did not have to match the natural colors of their motifs. With this contrast in balance and harmony to the impressionistic style, expressionists did not try to reestablish the individualism of each single object captured in their paintings, but by creating constructions of large contrasting shapes, they rather tried to overcome objectivism and distinctions between objects and subjects (2).

In detail, we allowed for a slight split in each era’s consistency regarding the, for some experts (2, 3, 4, 5), obvious, stylistic change within both. As a result, two sets for each era emerged, with the year 1884 marking the transition from “Early Impressionism” to “Late Impressionism” and the year 1910 separating “Early Expressionism” from “Late Expressionism”.

Styles of artists in the realm of ambiguity between these two eras, signifying commonalities and differences between them at the same time, are a main focus of the present study. This ambiguity represents a common problem in categorizing history in distinguishable eras. Because even though art historians like Coellen (2) or Landsberger (3) pointed out individual parameters on which to identify one of the two eras, the disagreement on the categorization of individual artists from between the two eras casts doubt on the validity of such parameters. In the current study, Cézanne, Gauguin, and van Gogh are chosen as three of the most prominent representatives of such artists without a clear assignment of all of their work to either Impressionism or Expressionism.

Paul Cézanne is a unique artist whose style is difficult to categorize and, in turn, different theories focus on different aspects of his work in doing so. Popular theories, for example, label Cézanne as the founding father of the post-impressionistic idea in France (5) or as an influence on Neo-Impressionism (6), which succeeded Impressionism and gave way to the expressionistic movement. Such conceptions, on the one hand, should make his work distinguishable from that of Impressionism in general, but, on the other hand, as a post-impressionist style similarities of his works to Impressionism are also possible if not likely. Such a categorization is supported by Cézanne’s reduced and less varying palette of colors and his stylized use of shapes in his paintings, which are all traits of a changing idea of Impressionism, focusing more on the essential characteristics of nature instead of its details (5). Therefore, the connection between Cézanne and Impressionism is regarded as undeniable (6) and even argued for by example of his prominent artworks containing the very same post-impressionist features ( 7).

Cézanne’s way of painting has also been described as extending the natural shape of objects and reducing the visual impression of reality to contrasts in colors and tones, which are rather expressionistic features (8). Other theories pick up on the visual features of Cézanne’s work, which were formerly regarded as post-impressionistic, and relate them even to cubistic conceptions (9, 10) differentiating the art historians’ approach in categorizing Cézanne even further. However, some of the art historians arguing for Cézanne’s stylistic variety also refer to him as an artist who deeply devoted his work to the ideas of Impressionism during his whole life (2, 5, 8). Regarding this devotion and besides the many stylistic ties Cézanne’s work was argued to reveal, a general rootedness of Cézanne in the impressionistic movement seems out of question.

When Gauguin started his career as an artist, he was inspired by Impressionism and even sent his paintings for a review by impressionistic societies (11). Due to the constant interaction between Gauguin and Impressionism at the beginning of his life as an artist, a basic impressionistic inﬂuence on his style cannot be denied (12). However, different art historians report an increasing change in Gauguin’s perception of arts, which led him away from his impressionistic roots and made him choose a stronger inspiration by Cézanne’s style (5, 12) from 1888 on. This would have eventually led him away from the original impressionistic style (11) and, like Cézanne, to adopt a more expressionistic approach in his paintings. One new feature emerging in Gauguin’s style during that time was a heightened density of colors, which Cézanne already used, reducing his palette to only a few tones of colors and thus giving his paintings much more expression than before (5, 12).

It is for such features and syntheses of colors and motifs that Gauguin’s art was later considered as being a precursor of modern art, which functioned as the transition from Impressionism to Expressionism ( 13). By including pictures from his memory and religious motifs, Gauguin developed a more fantastic and symbolic style representing a new relation between artist and nature, which was clearly in opposition to the neo-impressionistic movement of his days (5, 12). In sum, Gauguin’s style can be regarded as similarly varying as Cézanne’s: Being originally qualified as a member of impressionistic groups, a constant relation between Gauguin and Impressionism as such cannot be denied. However, as he clearly rejected the impressionistic approach in his later years by following admittedly pre-expressionistic conceptions, a similarly reasonable relation between Gauguin and the expressionistic movement exists positioning him right in the era of ambiguity investigated by the present study.

Regarding the art style of Vincent van Gogh, many Dutch painters, mainly naturalists, influenced his early work leading him to paint directly in and after nature (14). Besides Dutch artists, however, van Gogh was already interested in French painters at that time, such as Millet, who seemed to have had an impact on him (14). In line with the ideas of other favorite painters of his, van Gogh also tended to paint more caricature-like paintings of people instead of realistic ones ( 4, 14). Many sources, thus, agree that van Gogh’s interest in the early impressionistic period was not that strong and that he was rather skeptical regarding them (4, 5, 14).

In fact, van Gogh made no use of realistic coloring, but rather made abstract use of colors to realistically express his emotions and inner movements while seeing a natural scene, instead of representing the scene itself in every detail (2, 4, 5, 14). Therefore, van Gogh is argued to have taken major inspiration from other popular styles of his time, such as Cloisonnism, Neo-Impressionism, and Pointillism, which also reduced motifs to simple uses of colors (5, 14). Thus, van Gogh’s idea and approach highly resemble Gauguin’s, as both tried to convey their feelings as elicited by nature onto the canvas (2). Consequently, art historians account for van Gogh as a precursor of the expressionistic movement’s original ideas for the same reasons as they did for Gauguin ( 3, 13, 15, 16). Next to the expressionistic interpretations of his style, the ambiguity in van Gogh’s art leads to other classifications, for example, as a Post-Impressionist (5) or as an Impressionist per se, even though his style is unanimously regarded as one which led to the end of Impressionism (2, 4).

In the end, with most art historians approving the impressionistic influences on his early works, van Gogh can nevertheless be related to Impressionism. However, as a mutual influence between van Gogh and Gauguin also existed, relations to the latter’s art style must also be noted. With van Gogh adopting features of Gauguin and, in that way, indirectly of Cézanne, the account given for him as a pre-expressionistic painter also bears comparison to the former interpretations.

The general ambiguity underlying the styles of Cézanne, Gauguin, and van Gogh and the resulting connection between the three is elucidated further by the amount of times their works have been exhibited together and the impact these exhibitions had on the world of modern arts ( 13, 16, 17, 18).

### Previous Work on Visual Parameters of Artworks and Human Psychophysics

As artworks are deliberately made for being perceived by human observers in that they exhibit a quality that makes them "inherently interesting" (19), the investigation of how human perception is influenced by the artwork is an often studied field. Already in 1935, Buswell studied the directions of eye movements of participants looking at photographs of art (20). Since then, the field was more and more refined, for example, by studying the viewing behavior of special age groups (21). But the usefulness of recording eye-movement data to understand human perception of art is still not without doubt ( 22). Differences in the viewing behavior were found depending on the task participants got, with participants exhibiting longer fixations when asked for aesthetic quality of an image in contrast to when being asked for the content (23). This relates to the study of influences of bottom-up and top-down processes in the perception of art. A study conducted by Massaro et al. (24) showed how participants would rate an image, as well as the differences in exploration patterns in color, black-and-white, human and natural scenes by means of the influence of these variables on the eye-tracking behavior of participants. However, in addition, the influence of the content of images on viewing behavior is of interest. Serino and Villani (25) investigated gaze behavior on artworks showing individual movements or social interaction. Hayn-leichsenring, Lehmann, and Redies (26) found that statistical image properties can predict different art periods and the individual ratings by participants on beauty and aesthetic value of artworks. Mould, Mandryk, and Li (27) and Li, Mould, and Davies (29) used computer techniques to investigate the influence of non-photorealistic rendering on emotional responses, viewing behavior, the perceived aesthetic structure and response times of participants, respectively. For more on statistical image properties, see the review by Graham and Redies (19). Given that abstract artworks also elicit emotional reactions, Yanulevskaya et al. (30) used eye-tracking and computer vision techniques to investigate the emotional content of abstract artworks. Fuchs-Leitner, Ansorge, Redies and Leder (31) computed artwork's image salience in terms of local feature contrasts in color, luminance, as well as orientation and with these found salience effects by analyzing eye-movements. Moreover, they also found characteristic short-lived temporal profiles of these salience-driven effect on fixations.

As the formation of art categories and epochs is a fundamental aspect of perception or "Cognition as Categorization" (32), researchers are not only interested in the differences of artworks from different periods. To find out if canonical art periods can be seen as perceptually distinct, Wallraven, Cunningham, Rigau, Feixas, and Sbert ( 33) asked participants naive to art to cluster several hundreds of paintings based on their understanding of art style. Although some artists were clustered more consistently than others, participants clustered pieces of art into their canonically ascribed periods significantly more frequently than expected by chance. Additionally, the participants’ clustering reflected the transition of the art style during the impressionistic period. Participants showed surprisingly canonical categorizations, given that they were non-experts. They also used some low-level computer vision algorithms to try to categorize the artworks into different periods, but results showed that none of the low-level image properties correlated with human judgments. Regarding expertise, Augustin and Leder (34) using a sorting-for-similarity task found that experts processed art-works more in relation to style, whereas non-experts refer to criteria such as individual, personal feelings, in accordance with Leder, Belke, Oeberst, and Augustin ( 35).

On a more abstract level Leder, Belke, Oeberst, and Augustin (35) established their model on the perception of art, stating that the perception of art is dependent on an interplay between intrapersonal and exterior circumstances, some of which with a clear relation to visual parameters and style processing being essential (e.g., symmetry). The role of style in art has also been studied in paradigms of brief presentations, see Locher (36) for a comprehensive overview of paining gist, and: Bachmann and Vipper (37) found that at short presentation times artworks are more unstable, but sensitivity for style, e.g. abstract versus realism develops fast. M. Augustin, Leder, Hutzler and Carbon (38) used a similar paradigm, and found that at 10ms masked presentation, participants showed sensitivity for differences in artworks content, but not style; but already with 50ms masked presentation the style of the artworks (e.g. painted by van Gogh or Kirchner) affected evaluations of similarity of two simultaneously presented artworks. Nadal (39) gives an overview about studies dealing with neuroscientific approaches on human artwork perception, again some of which rely on visual properties.

### Present study

From these considerations, one can clearly see that it is an open question how well art styles are discriminated by parametric visual properties alone. While some of the mentioned characteristics in the debate, such as a reduced color palette, offer themselves to a more formal description, for instance, as a lower variability of colors used, others seem to be less easily represented by visual properties. Think of the naturalness versus abstractness of the motif as an example. Of course, it is conceivable that the corresponding characteristics are also reflected in visual properties, for instance, the distribution of spatial frequencies following or not following the typical distribution in natural images, it is also possible that some of these characteristics are not covered by any formal parameters at all or require human observers to bring the influence of such properties to life and effect (i.e., as a difference of coloration of an object in an image as compared to reality). Therefore, we used a set of paintings as training material and extracted whatever visual properties our deep neural networks extracted from these paintings and presented the resulting generated images to human observers.

In addition to the theoretical classification of styles, scientific measurements can nowadays be used to complement art historians’ endeavors. So far, these undertakings have for the most part been based on preconceptions about artworks’ particular inherent properties and, thus, represent only certain aspects of an artist’s or epoch’s entire style. Adequately relating artists to major artistic epochs, however, requires comparing the individual artist’s style as an entity with the respective era’s potential total style. Such comparison, as the diversity of theories in art history demonstrates, potentially transcends human intuitions and informed reckoning and instead demands for a new form of comparison, based on comprehensive and less assuming methodology. Those methods are now available. In the fields of empirical aesthetics and psychology of arts, we can use objective human behavior to test if different styles are discriminated, which does not require prior expertise on the part of the subjects about different art styles, and which does not require subjective evaluations of the artworks either. These fields and methods offer opportunities, which put art historical endeavors to an independent test. They could, thus, add valuable information to the investigations and put theories of art historians into perspective.

**Figure 1.**
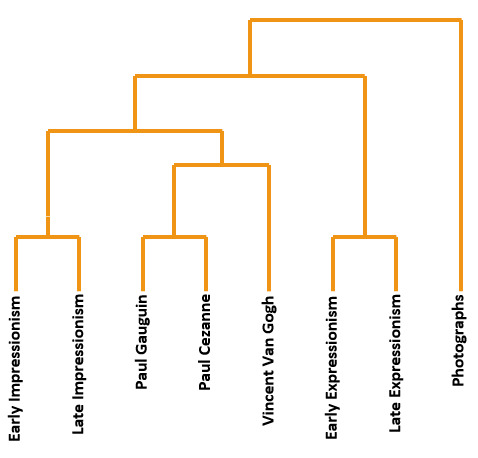
Clustering of art styles based on art historical theories.

For a better understanding of the data to be gathered by the following experiments, we constructed a theoretical hierachical clustering, based on the art historical relations between painters and epochs in our literature search and review above. This is presented in figure 1. For example, in our review above, we pointed out that van Gogh had demonstrated direct contact with Gauguin, and as the latter was also inspired by Cézanne, Cézanne’s style figured as one root of the stylistics shared by both van Gogh and Gauguin. In Figure 1, however, van Gogh is positioned further away from Cézanne than Gauguin, as van Gogh only got demonstrated inspiration from Gauguin and, thus, the influence of Cézanne was probably more remote and indirect for van Gogh than for Gauguin. Other relations in Figure 1 are even easier to understand, such as a closer relation or similarity between late and early phases of Expressionism than between the late phases of the historically preceding late Impressionism and early Expressionism. This relationship difference simply reflects the qualitative step of one style to the next implied by the usage of the different noun labels for the respective styles of Impressionism and Expressionism that is in contrast to the mere gradual degrees of difference expressed by the adjective qualifiers “early” and “late” for the different phases of the same styles.

In the following, we will construct similar hierachical clusterings from the data we gathered in our eyperiments. These clusterings depict similarity relations between styles, with clusterings closer and linked to one another sharing more characteristics than clusterings further apart or linked at more distant locations. Thus, we will have the possibility to compare the clustering based on the literature from the field of arts with clusterings based on quantitative data from our experiments. For example, in visual search, a higher similarity between the single outlier target image of one style (e.g., early Impressionism) and of the three distractor images of another style (e.g., late Impressionism) should slow down visual search for the outlier (1). Consequently, the comparison of both clusterings will function as a key hinge of the present study to compare the results to art historical literature and, thus, deliver mutual support or falsification for the fields’ individual theories versus the data’s classifications. We will perform a comparison of these clusterings in the discussion section of this paper.

## Methods

To investigate the taxonomy of art styles with quantitative methods, we used artificial neural networks to extract features and subsequently transform photographs into “generated images” from different epochs. This has the important advantage to abstract over the contents of the painting and being able to compare images of the same scene in different styles. These were then used in two psychological experiments with human participants. Humans should only show evidence of behaviorally discriminating between the different styles in these generated images if there were some underlying visual differences between the styles that were picked up by the neural networks.

### Stimulus Generation using CycleGANs

To have controlled stimuli in different art styles, we trained neural networks to apply an art style onto photographs. The goal was to have the same photograph painted in all art styles under investigation, such that the only distinguishing factor is the painting style without having to consider differences in the image content.

For the art style transfer we decided to use CycleGANs (40), since they can capture the similarities of a whole epoch and transfer the respective style on new images. We also considered other network structures, in particular Neural Style Transfer (41), but this algorithm is only able to transform the style of one image to another instead of the overall style of an epoch.

We used an already existing implementation of a CycleGAN which was written for transforming horses into zebras and vice versa^1^. We additionally added some data augmentation which makes the model train on random excerpts of the paintings to increase the size of our training data.

CycleGANs are a type of generative adversarial network (GANs) where two networks, one generator and one discriminator network, are trained simultaneously in a competitive setup (42). In our example, one can conceive of the generator network as an art forger who tries to create particularly good forgeries of paintings from the different epochs. In this example, the discriminator network would then be an art expert who has seen many real paintings from the epochs already and who has the task to discriminate the forgeries from the real paintings. During the training process, both parties improve, the generator gets better at creating realistic forgeries and the discriminator gets better at telling the false from the real paintings.

**Figure 2.**
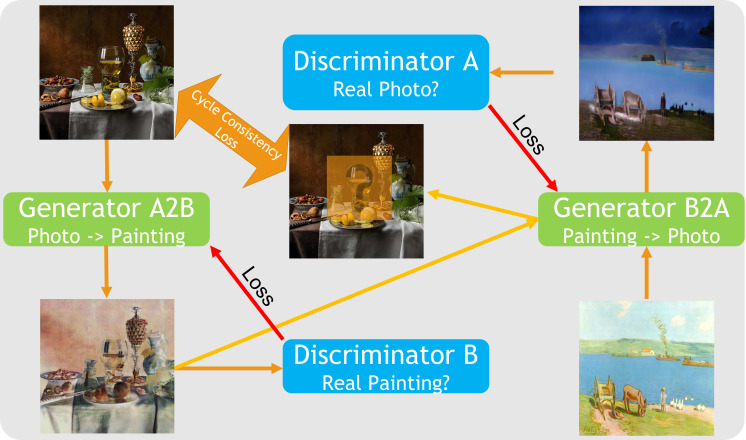
CycleGAN network structure.

The unique feature about CycleGANs is that this adversarial training process is performed in both directions (painting to photograph and photograph to painting) which makes it possible to use a cycle consistency loss. The idea behind this loss is that when transforming a photograph into a painting and then transforming this forgery back into a photograph, it should be as similar as possible to the original input. This is especially useful when there are no paired examples in the training set (40) as in our example where we do not have actual forged images based on the individual photographs we use for training. This means we have two generator networks, one of which learns to transform photographs into paintings while the other one learns to transform paintings into photographs. Additionally, we obtain two discriminator networks, one to recognize forged paintings, the other to recognize forged photographs (see Figure 2).

We trained our generator networks to output images of size 412 x 412 pixel which is the maximum size that still fits on a Nvidia Titan X Graphics Card with 12GB VRAM. Higher resolution images can be generated but with a quality tradeoff in the transformation since the neural network would have to be smaller.

**Figure 3.**
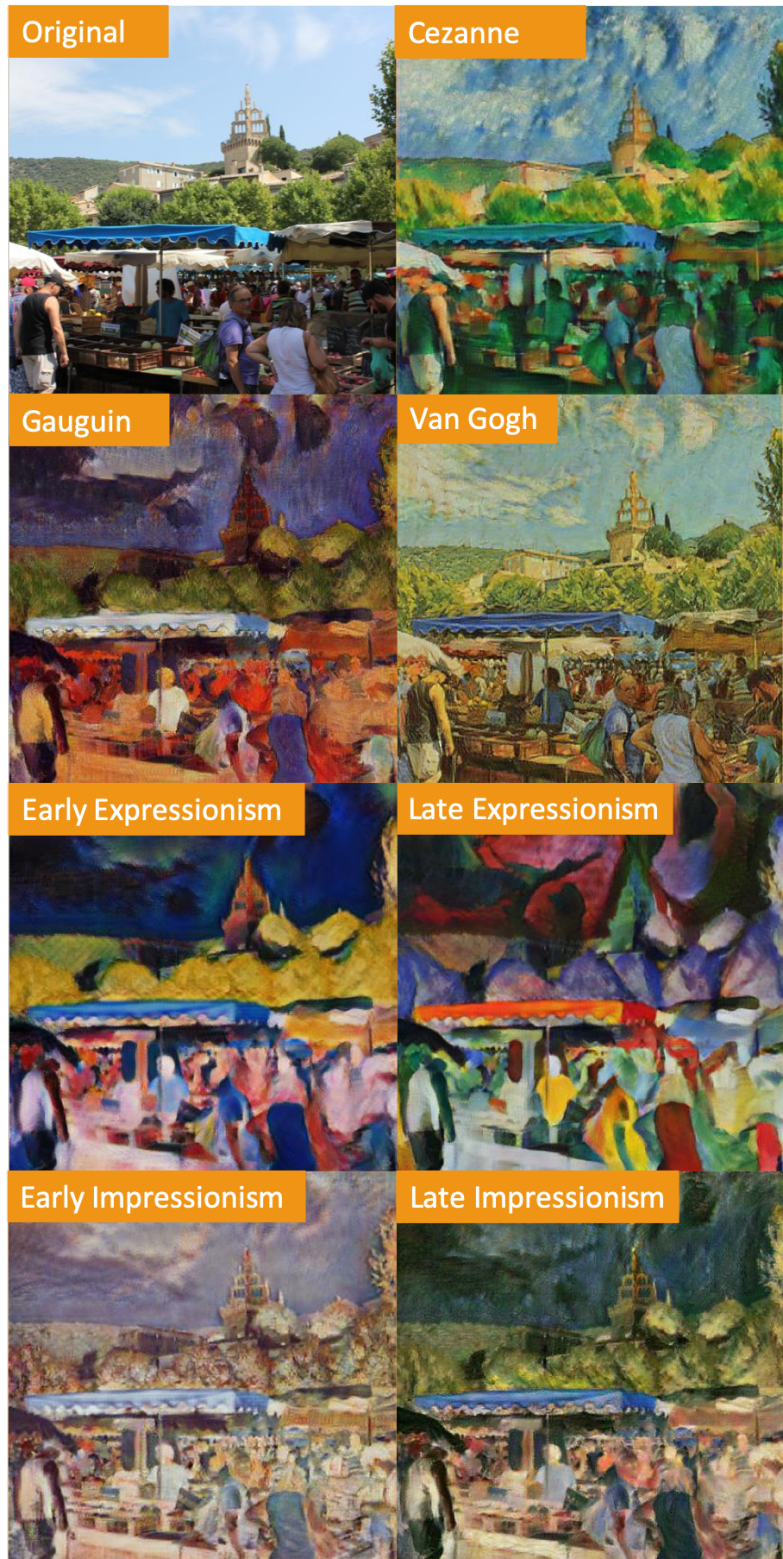
Example photograph (top left) transformed into the generated images of seven different styles using CycleGANs.

For training of the networks, we used two computers with Titan X graphic cards. Training of one network took two to three days. Overall, we trained at least four networks for each style. Styles that yielded results that looked very artificial to the human eye were trained more than four times. Characteristic acceptable generated images can be seen in Figure 3.

## Results

We conducted two experiments for this study. One singleton search experiment and one eye-tracking experiment. For both experiments, we used the same artificially generated images while the images used for the singleton search experiment are a subset of the images used for the eye-tracking experiment

### Experiment 1: Singleton search

**Figure 4.**
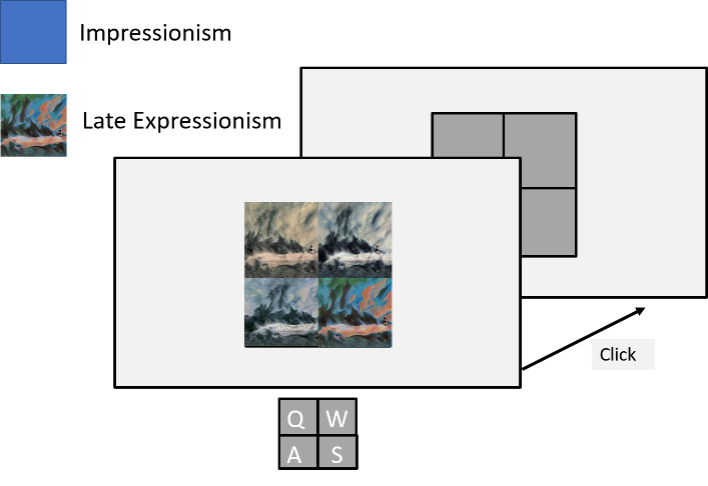
Singleton search experiment setup: The subject sees four images arranged in a grid. One stimulus is presented in one style and is the target, the others are presented in a different style and are the distractor stimuli.

During the singleton search experiment, per each trial, a subject saw four images arranged in a square pattern around the screen center (see Figure 4).

Each of the images had a size of 412 x 412 pixels and showed the same content. Three of the images were generated by networks of one style, the fourth was generated by a network of a different style. The three images of one style came from three different networks trained on the same dataset, such that they represented the style they were trained on with slight variations. These three images served as distractors while the image produced by the remaining network was the outlier target style. The subject’s task was to indicate the location of the target style. This was done by clicking one of four keys on a keyboard, each key representing the location of one image on the screen.

There was no time limit for giving a response. However, subjects were asked to respond fast and follow their first intuition. After a subject had given a response by pressing one of the four keys, the next image quartet was shown. After 56 trials each style combination had been shown once (once as target and once as distractor). The order of combinations as well as the selection of motifs was individually randomized for each subject. None of the motifs were shown twice to one subject. We presented four blocks of these 56 combinations to each subject, such that at the end of the session the subject had searched-for and thus selected, 224 different target images among its distractors. One session took between 10 and 20 minutes. For each trial, we recorded the chosen stimulus as well as reaction time.

We recorded data from 117 participants in total. Participants were recruited on the campus of the University of Vienna and were offered compensation in the form of cookies and snacks. To acquire this number of subjects we utilized several laptops which resulted in varying distances of the subjects to the screen, always keeping the keyboard close enough to be used comfortably. We used laptops with screen diameter of 13.3 and 15,6 inches and a screen resolution of 1920x1080 pixels. A quick look at the data gathered from our singleton search experiment shows the following statistics (see Table 1).

**Table 1:** Statistics from the singleton search experiment.

	Correct	Wrong
Mean	2.24	3.17
Median	1.61	2.29
Variance	4.42	8.79
No. Samples	17,621	6347
% Outliers	7.82	5.99

For the singleton search data analysis, we used reaction times and correctness of the participants’ choices as dependent variables. The reaction times were winsorized. Winsorizing is a data-processing step in which data deviating by more than +/-3.5 SDs from the mean is projected to the values corresponding to +/-3.5 SDs from the mean. Prior to analysis, we excluded fast guesses (i.e. trials with reaction times below 150ms) as they usually corresponded to accidental double-clicking.

**Figure 5.**
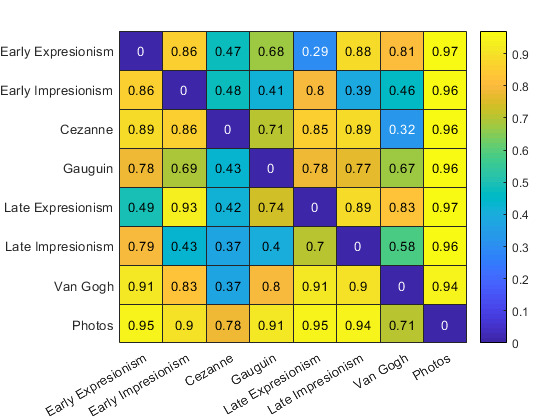
Average accuracy of participants relative to pairs of styles. Row labels denote the identity of the target stimulus, column labels denote the identity of the distractor stimulus.

**Figure 6.**
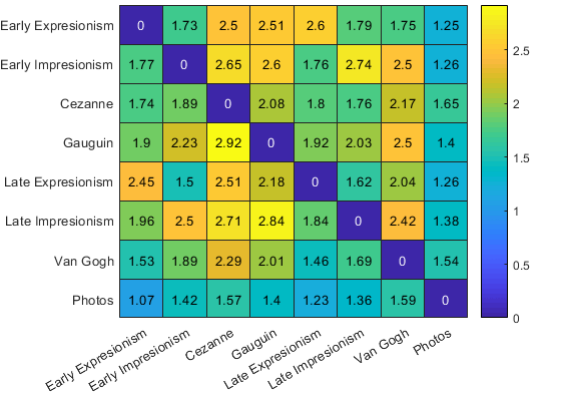
Average reaction times between styles in seconds. Row labels denote the identity of the target stimulus, column labels denote the identity of the distractor stimulus. Times are given in seconds.

We visualized the accuracy in percent (Figure 5) and reaction time in seconds (Figure 6) between styles in a matrix. The row labels denote the identity of the target stimulus style while the column labels denote the identity of the distractor stimulus style.

**Figure 7.**
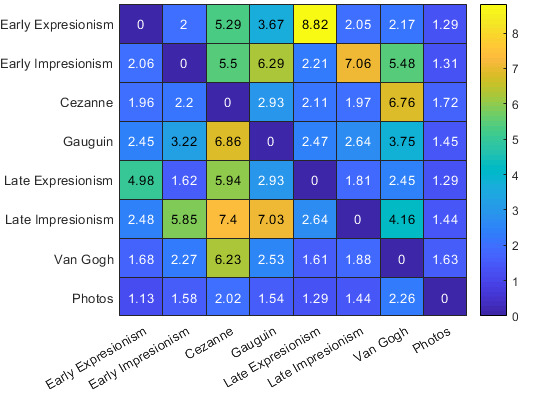
Effective reaction times between styles. Row labels denote the identity of the target stimulus, column labels denote the identity of the distractor stimulus.

Effective reaction time, which is a joint performance measure based on reaction time and accuracy, can be calculated as the quotient of reaction time divided by accuracy. This creates a matrix that penalizes long reaction times with low accuracy while reaction times associated with high average accuracy are left unchanged. This operation leads to the matrix presented in Figure 7.

**Figure 8.**
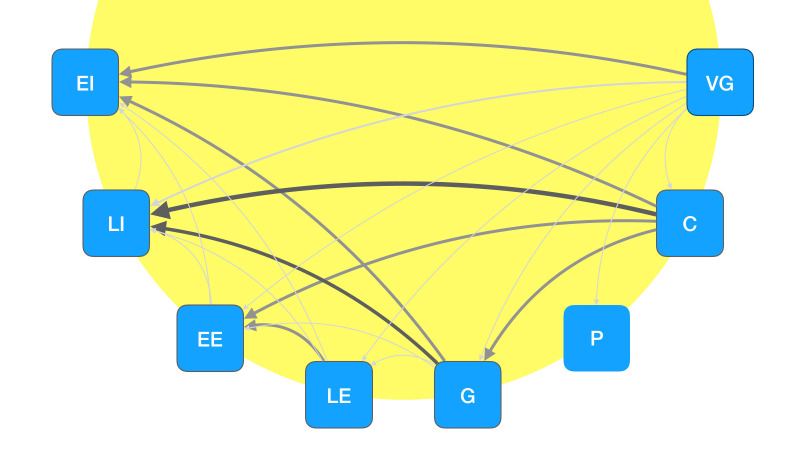
Transitive relationship between styles. The direction of arrows denotes that a strict hierarchical relation exists between styles. The thickness of arrows denotes the difference between a style serving as distractor and as target when compared to another style. EI: early Impressionism; LI: late Impressionism; EE: early Expressionism; LE: late Expressionism; G: Gauguin; P: Photography; C: Cézanne; VG: van Gogh.

**Figure 9.**
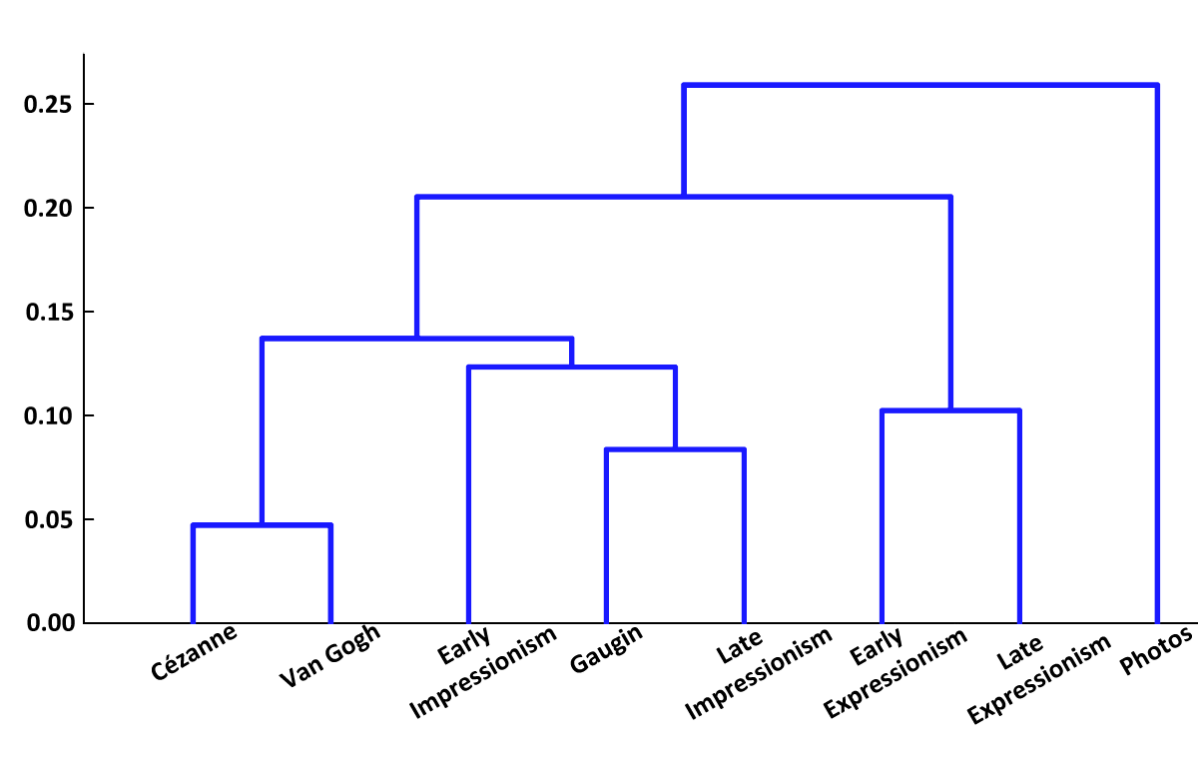
Hierarchical clustering of styles. The length of the lines denotes the stylistic distance.

Having computed the quotient, we were able to compute the element-wise inverse of the penalized matrix and calculate the distance between styles. Using average linkage, reaction time and accuracy data provided enough information to create a hierarchical clustering of styles. Figure 8 depicts the transitive relationships between styles, and the resulting hierarchical clustering is visualized in Figure 9.

As can be seen in the matrices, low accuracy values between styles correlate with high reaction times between the same styles. This indicates that there is a metric with which styles with certain characteristics take longer to identify and are less likely to be discriminated correctly. We took this characteristic to be an index of the similarity of the visual appearance of a style.

Further, the transitive nature of our reaction time-accuracy quotient shows that there is a profound difference in stylistic distances between styles: For example, while images in the style of van Gogh and Cézanne are easily distinguishable from all other styles, they are not easily distinguishable from one another (see Figure 8).

### Experiment 2: Eye-Tracking

**Figure 10.**
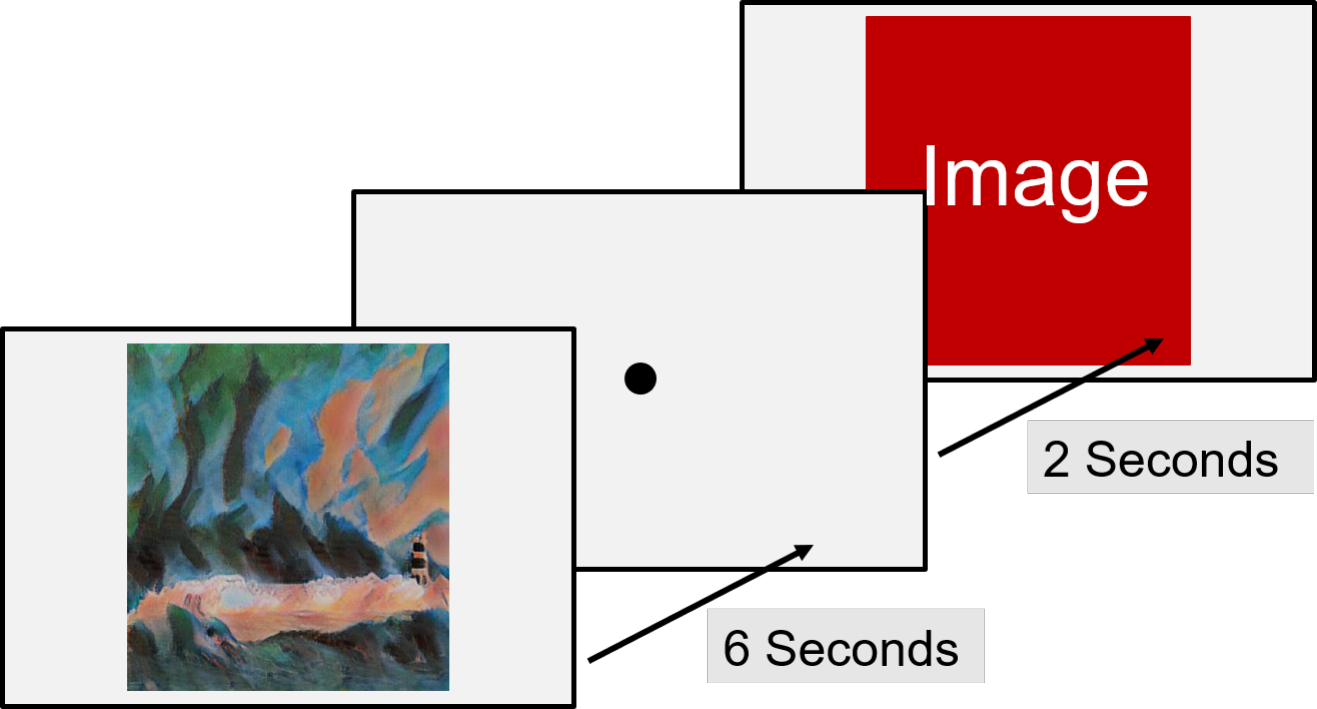
Eye-tracking experiment setup.

The eye-tracking experiment used a free viewing task in which the subject had no other instructions than to look at the images presented on the screen. Each image was shown for 6 seconds, after which a fixation cross was shown in the center of the screen (see Figure 10). Subjects were asked to focus their gaze on the fixation cross to ensure homogeneous initial conditions for every stimulus viewed by the subject.

After 60 trials, the eye-tracker was re-calibrated to compensate for a loss of accuracy over time and subjects were able to rest their eyes. The images were shown to the subjects in random order. Each block contained one image of each style. Each image motif was only shown once meaning that no subject saw the same motif in more than one style. Overall, each subject was presented with 30 images per style. This makes 240 images overall. The whole experiment took around half an hour.

The experiment was implemented with Psychtoolbox and MATLAB. Eye movements were recorded with an EyeLink 1000 Plus eye tracker. The subjects viewed the stimuli from a distance of 60cm, placing their head on a chin rest, on a screen of size 1280x1024. We measured 64 subjects in total.

**Figure 11.**
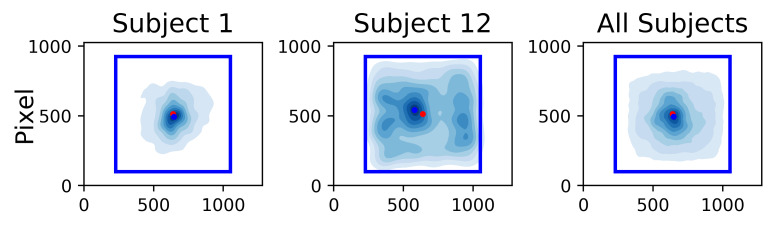
Fixation density maps for single subjects (left and center). Fixation density map over all subjects (right). Blue lines mark the size of the image on the screen in pixels (824x824). The axes lengths correspond to the overall screen size. The red dot marks the screen and stimulus center. The blue dot marks the point of the highest fixation density.

For the analysis of the recorded eye-tracking data, we investigated fixations on the images. Disregarding the art style, we can make out general patterns in the fixations such as a higher density of fixations at the center. Figure 11 shows the fixation density maps of two subjects (left and center) as well as the density map over all subjects. One can see that there are some inter-subject differences in the amount of visual exploration on the stimulus area.

**Figure 12.**
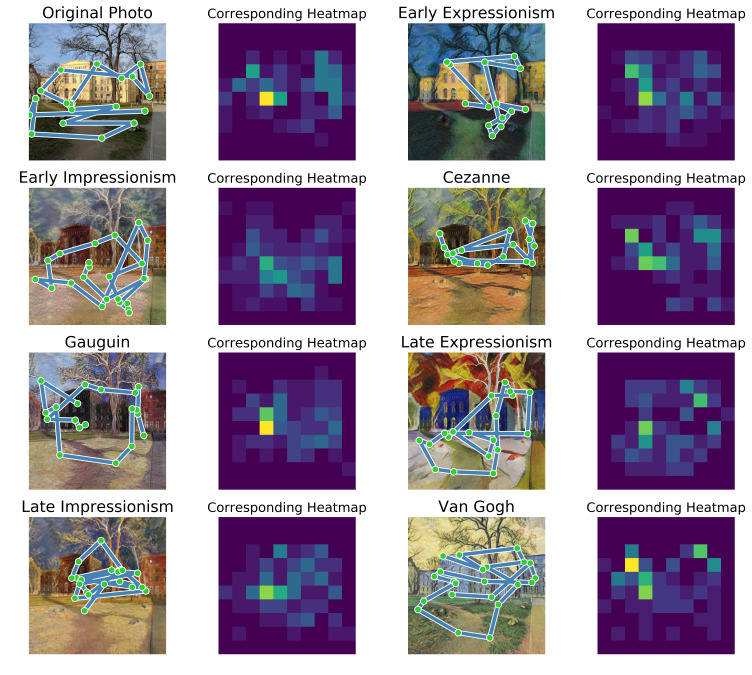
One photograph and its transformations in all seven art styles with the corresponding eye-tracking data. Columns 1 and 3 show the stimuli with a gaze path of one example subject. Columns 2 and 4 show the corresponding normalized heatmaps of fixations by all subject that were presented with the respective image.

Since each photograph was presented in eight variations (seven art styles plus the original) to different subjects, we can now compare the fixations between art styles disregarding the content of the image. In Figure 12, one can see one photograph (top right) in its eight variations and the corresponding eye-tracking data. Each image has the gaze of one example subject drawn on top of it to give an impression of the amount of visual exploration that could be performed in the given time.

**Figure 13.**
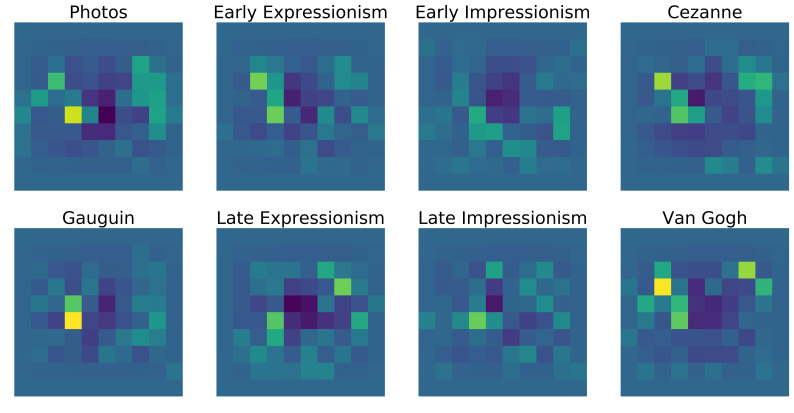
Normalized heatmaps of all fixations on the example images shown in Figure 12 after subtracting the grand total bias.

Every subject would only see the image content in one style to avoid effects of familiarity in the fixation patterns. This means that each of the images shown in Figure 12 was seen by a different sub-group of subjects. Due to this, we only have an average of 129 fixations on each image (on average 16 fixations from each subject that saw the image). Therefore, we decided to sort all fixations into a 10 x 10 grid which was then used for the comparison between art styles. We think that this size provides a good tradeoff between spatial granularity and data sparsity. One entry in the matrix corresponds to an area of 82.4 x 82.4 pixel on the screen. Because some heatmaps contained more fixations than others, we normalized them such that the integral over them equals 1.0. Additionally, we subtracted the grand total bias to extract only style-specific effects and to avoid misleading correlations. The heatmaps from Figure 12 after normalization and subtraction of the grand total bias can be seen in Figure 13.

**Figure 14.**
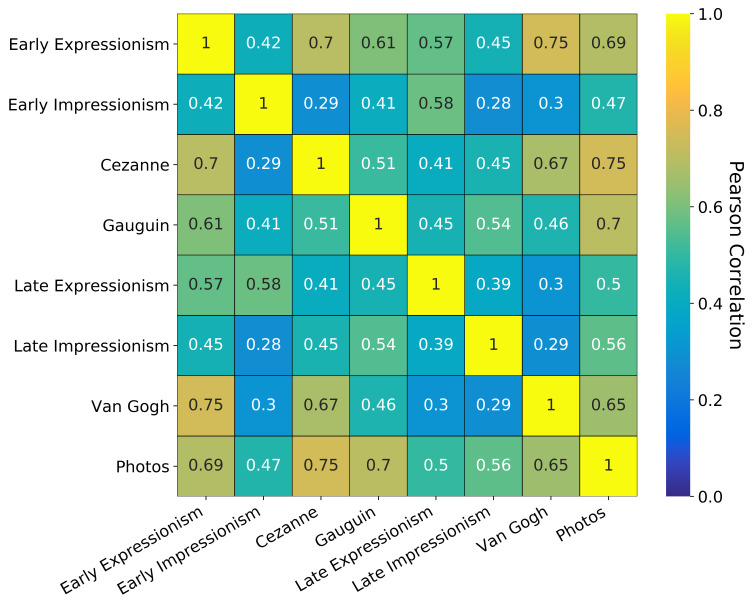
Correlation matrix of Pearson correlations between fixation heatmaps of the example image shown in Figure 12.

To compare the fixations on the different styles, we now transformed the heatmaps shown in Figure 13 into 1D vectors and calculate the Pearson correlation coefficient and the corresponding p-value for each combination. The resulting correlation matrix for the example image shown in Figure 12 can be seen in Figure 14.

**Figure 15.**
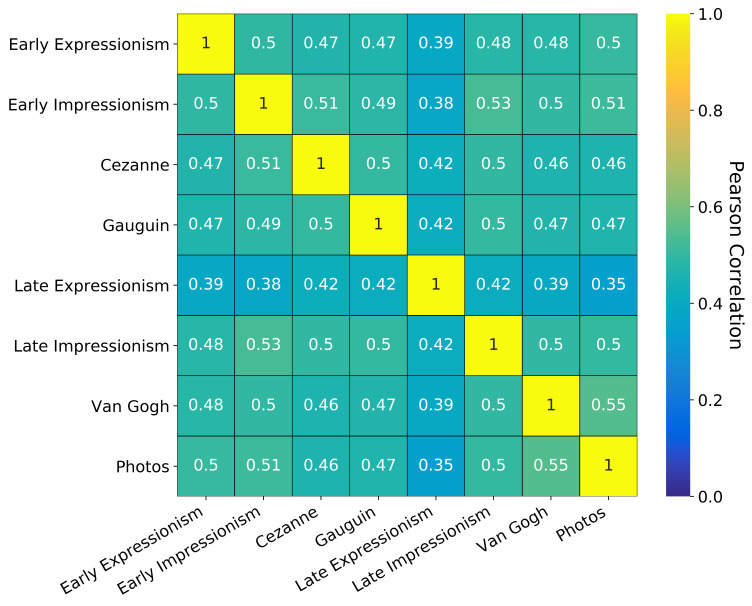
Correlation matrix of fixations on all images in the eight different styles.

**Figure 16.**
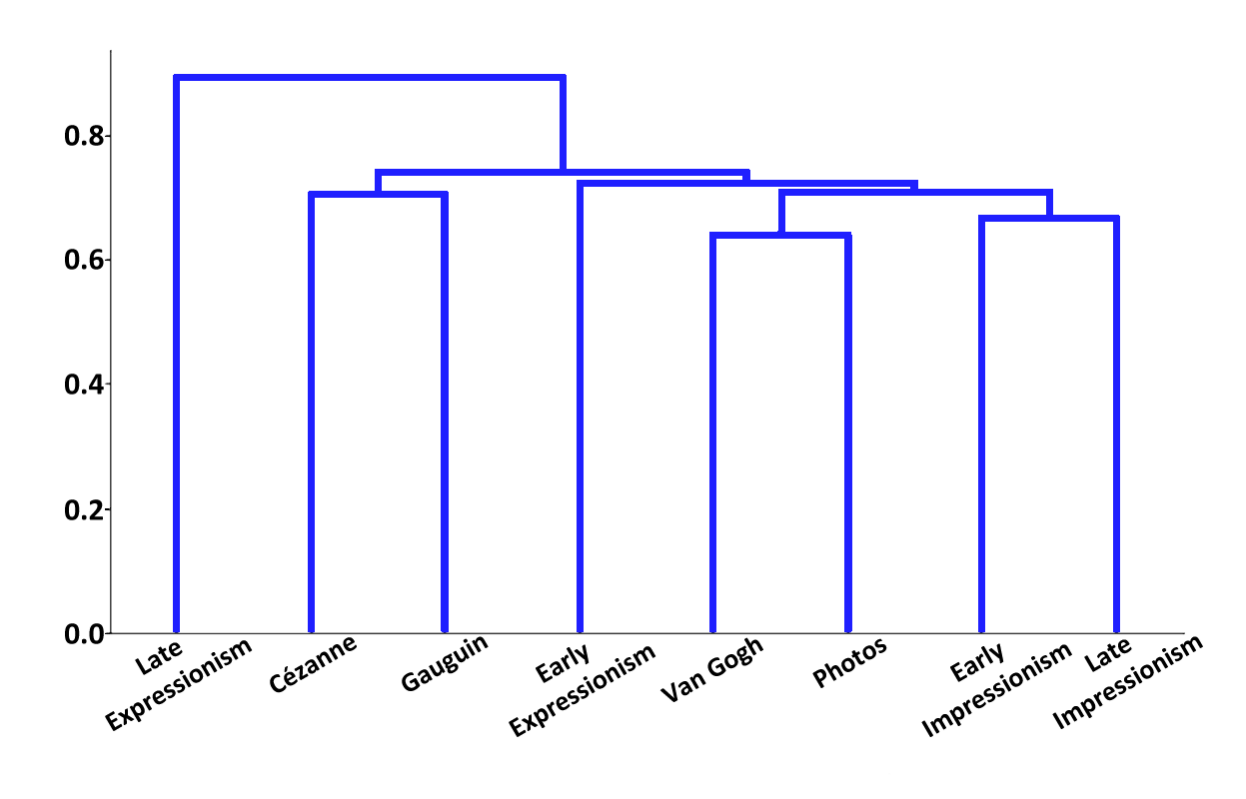
Hierarchical clustering constructed from the correlation matrix shown in Figure 15. It shows the clustering of styles based on the accumulated fixations.

To obtain overall correlations between the different styles, we average the correlation matrices of all 240 images in their eight styles (Figure 15). Finally, we used this correlation matrix to calculate a hierarchical clustering of the eight styles (Figure 16). To create the clustering, we used average linkage.

**Figure 17.**
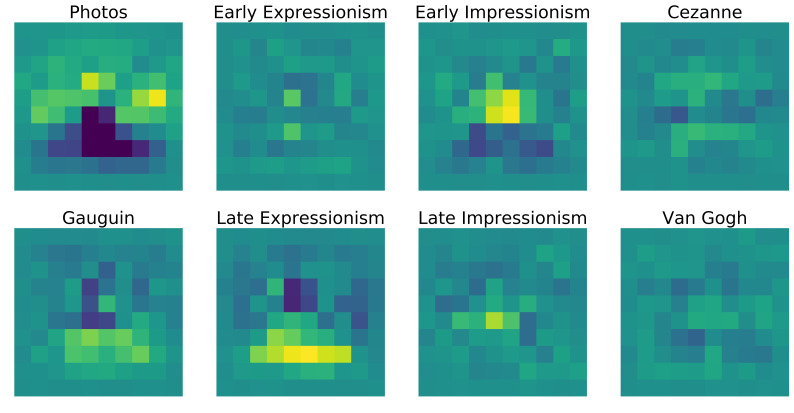
Accumulated fixation heatmaps over all 240 images in each style. The heatmaps are normalized and the grand spatial bias is subtracted.

In the correlation and the clustering, one can see that there are only marginal differences between the eight styles. Even though there are differences in the correlations between different styles on the individual image basis (figure 14), these differences average out over all images. The image content dependent differences outweigh the overall differences between the fixations on the image styles. However, when looking at the accumulated heatmaps of all images (figure 17) one can see some overall differences between the styles that get lost in the averaged correlations.

## Discussion

Having analyzed our data, we now turn towards integrating the results into the context of empirical aesthetics and art history.

From our singleton search study, it was possible to construct a hierarchical clustering resembling differences discrimination ability between art styles. We see these differences as a reflection of aesthetic differences between art styles: as some discriminations were fast and effective, we conclude that the parametric differences between styles are mainly based on the visual differences and similarities between the coloring, brightness, and an overall impression, such as quality of rendition. These visual properties then allow a discussion of the present results once in terms of the study’s art historical background as well as behavioral studies investigating bottom-up and salience effects in the perception of artworks (34, 36).

When comparing the hierarchical clustering obtained from our singleton search study to the hierarchical clustering we created based on literature from art history, a general similarity concerns the clustering of the two phases of Impressionism and that of the two phases of Expressionism: Both eras’ early and late periods are correlated with each other, proving that already on a superficial level the respective era’s characteristics are easily detectable and are reflected in measurable behavioral differences. Such a correlation furthermore argues for a consistency of these characteristics throughout their development and, in turn, for an obvious contrast between the impressionistic versus the expressionistic use of colors and style of depictions.

In addition, consistency is also found in terms of the individual artists’ ambiguity, especially with van Gogh and Cézanne being clustered together in one clustering rather than with one of the two eras. Considering the previously analyzed art historical theories, however, such a clustering as well as the clustering of Gauguin with late Impressionism was not unexpected, as there are theoretical reasons for these correlations. However, one should note that probably not all characteristics advocated in theories on art styles are covered by our approach. In fact, in an experimental setting such as our singleton search, the determining properties of the art styles are probably mostly the visual properties on which the viewers based their decisions. Regarding the correlations between styles now being based on characteristics such as coloring, brightness, and quality of rendition, the present correlations seem to make sense as Gauguin is characterized by dark tones similar to the general tone of colors in the images generated by the impressionistic networks. Both styles are also still quite realistic in their structures, which could have led the viewers to see the present correlation between them. The same approach might account for the correlation of van Gogh and Cézanne, as they are the only styles depicting realistic structures in brighter tones and, thus, seem to be correlated on a merely superﬁcial level for the viewer.

Further support for the impact of the style of depiction on the participants’ selection is provided by the study of Augustin and Leder ( 34) in which the authors found that participants untrained in arts divided a selection of different paintings foremost according to their style of depiction, with realistic and abstract styles as the main criteria for divisions (34). Moreover, they name the difference between structured and geometric versus expressive and dark paintings as the second most important dimension for untrained participants. In the investigation of different research findings on the so-called “painting gist” by Locher (36) researchers found that superficial properties affect the perception of an artwork after very short presentation times ( 36). A study by Locher and Nagy (43) revealed that untrained participants were also able to categorize realistic and abstract paintings based on their respective pictorial balance after short exposures, underlining the immediate effect of a painting’s superficial content on the viewers’ perception. Furthermore, Cupchick and Berlyne ( 44) found comparable reaction times for the effect of the inherent order and structure of a painting’s properties on untrained participants. With no time limit given in our singleton search, a similar effect for the properties of the generated images seems plausible, thus giving an explanation for the originally unexpected correlation of Gauguin and early Impressionism: not only do both art styles represent a more realistic approach, but both are also foremost characterized by dark tones. Gauguin and early Impressionism thus converge on the first two dimensions identified by Augustin and Leder ( 34). More validation of Augustin and Leder’s (34) results to our study is granted by the fact that the same explanation would apply to the positive correlation of van Gogh and Cézanne: both styles are predominantly characterized by more structured content and brighter tones, thus corresponding to two of the dimensions responsible for categorizations given by Augustin and Leder (34).

Importantly, in these studies stimuli often had been presented under specific time limits, with Cupchik and Berlyne (44) and Locher and Nagy (43) presenting their stimuli under tachistoscopic presentation times of just a few milliseconds to investigate the minimum stimulus duration necessary for detection and recognition. Our singleton search experiment, on the other hand, additionally required decision making and further motor acts, which accordingly resulted in longer reaction times than in the aforementioned studies. Regarding art history, however, these scientific explanations also are critical from an art historical perspective: the dark tones in the images of early Impressionism might be responsible for their correlations with the art style of Gauguin, which are, however, only one hue of the otherwise rich palette of impressionistic colors and, thus, eventually do not represent the styles’ “true” properties. This distortion, however, does not exist in the correlation of van Gogh and Cézanne and, thus, leads us to the conclusion that, regarding their visual properties, both styles show characteristic similarities, which might account for a similar understanding of the impressionistic idea by both van Gogh and Cézanne. Even though our research did not indicate that van Gogh and Cézanne ever met and, therefore, we did not assume a direct correlation, with respect to the present ﬁndings we can at least further argue for the superﬁcial impact of the artistic idea mediated by a go-between (in this case, Gauguin), which two artists can share, regardless of personal inﬂuences on each other. The general correlation of the three individual artists with the impressionistic sets can, thus, nevertheless account for the frequently joint grouping of Cézanne, Gauguin and van Gogh under the label of post-impressionistic painters, giving more weight to the artists’ actual historical disposition and its influence.

Finally, it is worth mentioning that the greatest distance between styles is that of original photos and any other art style. This is not surprising, as the art styles considered in our work do not have the explicit feature of trying to be as realistic as possible. This maximum distance between photos and all other art styles yet reaffirms that our methodology applied in our singleton search experiment gives insight about the aesthetic relationships between art styles.

Our eye-tracking experiment did not deliver generalizable insight about the correlations between the different art styles. Art historically interesting analysis of the present results might be made about individual images, like the correlation between an image of Figure 12 in the style of van Gogh and the same image in the style of Cézanne, as well as the accumulated heatmaps for the same image in both styles in Figure 13: the respective accumulated heatmaps of both styles reveal similar points of fixation by the participants, with fixations in the van Gogh image apparently being more intensely fixated versions of the fixations in the Cézanne image. When comparing these heatmaps to the underlying images, these fixations focus on color contrasts between yellowish and blueish tones. Interestingly, it is these tones that define the style of van Gogh's later works, which were used to train the networks on because of their recognizability. Contrasts between yellow and blue, warm and cold colors were also largely used by Cézanne and, in some of his rather abstract works, used to facilitate recognition of the images' otherwise highly abstracted objects. In this regard, the styles’ accumulated heatmaps for individual images reveal an effect of the respective styles' defining characteristics on the participants' viewing behavior and on the relation between both styles. Therefore, these particular results would further support our interpretation of the singleton search results: even though no direct exchange between van Gogh and Cézanne can be found in art history, the artists' similar degree of devotion to a more general artistic idea might have led to a similarity in their stylistic representation of this idea.

The nature of the artistic idea underlying van Gogh’s and Cézanne’s art style furthermore seems to resemble the early expressionistic idea when regarding the high correlation between the images of the two individual art styles and early Expressionism: when again considering the accumulated heatmaps for one image, the areas of the most intense fixations for the individual artists’ styles match the areas in the early expressionistic style. In addition, the color contrasts causing these fixations correspond to the general tones of colors in the art styles of van Gogh and Cézanne: fixations on warm and cold contrasts and blueish and yellowish contrasts are easily detectable (however, for a discussion on aesthetic effects see Specker et al. (45). These contrasts, however, are more pronounced in the early expressionistic rendition of the image and less intense in the van Gogh and Cézanne rendition. This might be due to the respective styles' use of colors and contrasts, but the similarity in attraction becomes notable for this very reason: with the aim of our study to investigate new relations between individual artists and artistic eras, the correlations between the different versions of the individual image would lead us to the assumption that the tones and contrasts as established by both Vincent van Gogh and Paul Cézanne pioneered and influenced the succeeding expressionistic use of colors to considerable degree.

Not only would such an interpretation of individual images and their correlations from our eye-tracking experiment strengthen art historical theories but they would also seem to be in accordance with other studies. The correlation of van Gogh and Cézanne was already discussed in the light of the singleton search experiment, with studies by Augustin and Leder (34), Locher and Nagy (43) and Cupchick and Berlyne ( 44) substantiating the impact of the two art styles’ most prominent properties. The same properties could now be used to explain parts of the results of our eye-tracking experiment, while for the respective example, also making a correlation with early expressionistic styles plausible. The impact of colors and contrasts on eye-movements has already been studied (31): In their eye-tracking study, Fuchs-Leitner et al. investigated the “salience model” predicting the likelihood of fixations in paintings’ salient areas. Next to depictive paintings, the study’s stimuli also included artworks by Cézanne and abstract artworks thus resembling our set of stimuli and also meeting the previously discussed categorization criteria of realistic versus abstract styles. The results of their free-viewing task revealed a similar viewing behavior of untrained participants in terms of mean number of fixations and fixation duration for Cézanne and abstract paintings and are therefore in accordance with the general structure of the heatmaps discussed for Cézanne and early expressionistic images. More to the point, Fuchs-Leitner et al.’s analysis in light of the salience model showed that color and orientation contrasts functioned as the main predictors for the salience of the fixated areas, with the probability of local fixations being higher than chance level (31). Regarding these results in light of the interpretation made for the respective eye-tracking results of van Gogh and Cézanne, we can now assume that the similarity in salient color contrasts of Cézanne and early Expressionism attract the viewing behavior for both styles in a similar bottom-up way and are thus responsible for the similar viewing patterns in the different renditions of the same image. Furthermore, as all three art styles exhibit highly salient contrasts of the same colors in this example, the present results would support the theory that the art styles of van Gogh and Cézanne, at least in terms of the use of salient color contrasts, served as an inspiration for the art style of the subsequent era of Expressionism.

Another interesting finding in light of art historical theories can be found in the correlation between the image in Figure 12 in the style of Gauguin and its original version: both heatmaps show similarities in their fixations even though no direct similarity in colors or contrasts exists between the two images. In fact, the Gauguin rendition rather depicts the negative image of the original photograph. Besides these differences, however, no alteration in the original version’s structure or shapes is detectable, thus arguing for the inherent order and structure of both images being the cause for the correlation. As was already shown in the discussion of our singleton search results, previous findings highlight these properties’ importance for perception in a similar manner and would gain further validity with the implications of the individual images’ heatmaps (34, 44). In turn, the negative image of the original photograph was also present in the impressionistic rendition and thus gains further credibility for being the artistic tool causing the correlation not only in our singleton search but also for the individual image in our eye-tracking experiment. While the art style of early Impressionism could be correlated with the one of Gauguin in our singleton search, the art style of late Impressionism becomes more important for the present example. Additional analysis of other images from our eye-tracking experiment would probably divide the network of relations between the various art styles and the properties responsible even further.

The discussed implications of our eye-tracking experiment nevertheless need to be interpreted with caution. When we search for similar correlations between the accumulated heatmaps of all the images used in our eye-tracking experiment, the similarities in structure and intensity of fixations is no longer existent. The accumulated heatmaps’ patterns would even argue for different interpretations than the individual heatmaps: Gauguin’s art style would resemble the late expressionistic rendition of images, while van Gogh’s and Cézanne’s art style would rather correlate with the late impressionistic and early expressionistic renditions. These correlations, however, could be based on minor similarities between the otherwise too sparsely distributed heatmaps. Accordingly, neither transfer nor generalizability of the individual images’ implications took place and, therefore, did not lead to a reasonable comparison of hierarchical clusterings with art historical theories. Due to the small number of fixations for each image, the heatmaps are too different and content and subject-specific fixations outweigh style specific fixations. This might be different with a larger number of subjects. There might also be more evidence for behavioral discrimination if style specific content-differences were allowed. In general, one needs to keep in mind that this study only compared art styles on a parametrical visually basic level, accounting for colors, brush strokes and structure, explicitly disregarding content. Even though this method proves that visual properties are meaningful in discriminating styles even for laypeople as our subjects, as art styles are not solely defined by the methodologies the artist used to create these artworks but also by the content they display this cannot be seen as a complete account of the artist or art style.

Overall, we can say that our study showed that art styles can be classified using quantitative methods. In our setup, a simple singleton search experiment sufficed to cluster art styles into hierarchies similar to those created by art historians. Our eye tracking study did not reveal any meaningful insights. However, this might originate from our experimental setup and the sparsity of fixations we could collect through that and does not rule out the possibility to use eye movements to distinguish different art styles.

### Ethics and Conflict of Interest

The authors declare that the contents of the article are in agreement with the ethics described in http://biblio.unibe.ch/portale/elibrary/BOP/jemr/ethics.html and that there is no conflict of interest regarding the publication of this paper.

### Acknowledgements

This paper is based on a study project conducted at the University of Osnabrück, Germany. Also involved in this project were Peter Naeve and Falk Heuer whom we would like to extend our gratitude for their contribution.

We thank Tobias Schöberl and the Faculty of Psychology at the University of Vienna for supporting us with eye-tracking equipment and the possibility to hand out credits to students for participating in our experiments.

## Footnotes

https://github.com/xhujoy/CycleGAN-tensorflow
